# Evaluation of prostate cancer detection using micro-ultrasound versus MRI through co-registration to whole-mount pathology

**DOI:** 10.1038/s41598-024-69804-7

**Published:** 2024-08-14

**Authors:** Jake Pensa, Wayne Brisbane, Adam Kinnaird, David Kuppermann, Griffith Hughes, Derrick Ushko, Alan Priester, Samantha Gonzalez, Robert Reiter, Arnold Chin, Anthony Sisk, Ely Felker, Leonard Marks, Rory Geoghegan

**Affiliations:** 1 Department of Bioengineering, University of California Los Angeles, Los Angeles, USA; 2 Department of Urology, University of California Los Angeles, Los Angeles, USA; 3 Center for Advanced Surgical and Interventional Technology, University of California Los Angeles, Los Angeles, USA; 4 Department of Urology, University of Alberta, Edmonton, Canada

**Keywords:** Co-registration, Image reconstruction, Micro-ultrasound, MRI, Prostate cancer, Whole-mount pathology, Biomedical engineering, Prostate cancer, Magnetic resonance imaging, Ultrasonography

## Abstract

Micro-ultrasound has recently been introduced as a low-cost alternative to multi-parametric MRI for imaging prostate cancer. Early clinical studies have demonstrated promising results; however, robust validation via comparison with whole-mount pathology has yet to be achieved. Due to micro-ultrasound probe design and tissue deformation during scanning, it is difficult to accurately correlate micro-ultrasound imaging planes with ground truth whole-mount pathology slides. In this study, we developed a multi-step methodology to co-register micro-ultrasound and MRI to whole-mount pathology. The three-step process had a registration error of 3.90 ± 0.11 mm and consists of: (1) micro-ultrasound image reconstruction, (2) 3D landmark registration of micro-ultrasound to MRI, and (3) 2D capsule registration of MRI to whole-mount pathology. This process was then used in a preliminary reader study to compare the diagnostic accuracy of micro-ultrasound and MRI in 15 patients who underwent radical prostatectomy for prostate cancer. Micro-ultrasound was found to have equivalent performance to retrospective MRI review for index lesion detection (91.7% vs. 80%), while demonstrating an increased detection of tumor extent (52.5% vs. 36.7%) with similar false positive regions-of-interest (38.3% vs. 40.8%). Prospective MRI review had reduced detection of index lesions (73.3%) and tumor extent (18.9%) but improved false positive regions-of-interest (22.7%) relative to micro-ultrasound and retrospective MRI. Further evaluation is needed with a larger sample size.

## Introduction

Prostate cancer is the most common malignancy and the second leading cause of cancer death in men in the United States, with an estimated 300,000 new cases and 35,000 deaths in 2024^1^. Whereas clinically significant (Gleason grade 3 + 4 or higher) prostate cancer can be deadly, patients with low grade clinically insignificant prostate cancer (Gleason grade 3 + 3 or lower) have less than a 2% risk of cancer specific mortality 10 years after diagnosis^2–4^. Consequently, accurate cancer staging is vital for disease management as overtreatment of low grade cancer can result in significant decreases in patient quality of life and increased healthcare expenditures, whereas undertreatment of aggressive disease can be fatal^2^.

The conventional diagnostic pathway utilizes an ultrasound (US) guided systematic biopsy following an elevated value on a prostate-specific antigen (PSA) screening test^5^. While US allows for general gland visualization, it has poor sensitivity and specificity for tumor detection^6^. In contrast, multi-parametric magnetic resonance imaging (mpMRI) has high sensitivity (86–92%) and specificity (60–83%) for identification of prostate cancer index lesions^7–9^. Index lesions are defined as the largest and most aggressive tumor in a prostate, the size and grade of which are often used to decide the appropriate treatment pathway^10^. Consequently, current recommendations include an MRI-guided biopsy or more commonly: MR-US fused biopsy^11,12^. For fusion biopsies, a pre-biopsy mpMRI identified region-of-interest (ROI) is fused to a live US scan allowing for US guided targeted biopsy of an MRI ROI^13^. This targeted approach improves cancer detection, however, mpMRI is resource intensive, has substantial inter-observer variability, can still fail to detect 10–15% of clinically significant lesions, and tumor volume estimates can be low as 1/3 of the true volume^8,14–16^. In an effort to resolve these issues, a low-cost high resolution ultrasound technology known as micro-ultrasound (microUS) (ExactVu, Exact Imaging, Ontario Canada) has recently been introduced. This technology may enable direct identification of cancerous tissue on live US imaging, potentially mitigating the need for a pre-biopsy MRI.

**Figure 1:**
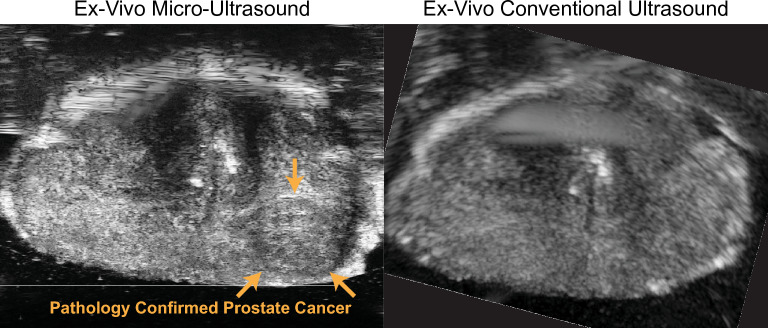
Comparative images between microUS (left) and conventional US (right) acquired in a custom-built ex vivo scanning system^20^. A WM confirmed lesion is visible on microUS only (orange arrows).

MicroUS uses a 512-element linear array 29 MHz transducer with inter-element spacing of 90 µm based off of the work of Foster et al.^17^. This allows for an axial resolution of 70 µm in proximal regions of the prostate, encompassing much of the peripheral zone, which also accounts for approximately 70% of all prostate cancers^17,18^. To image deeper in the prostate the microUS system uses a distance-based beamforming design that images more distal regions of the prostate at lower frequencies creating image bands of differing axial resolutions (70 µm [proximal]–100 µm [distal]) that comprise the full B-mode image. This approach allows for an imaging depth of 6 cm at the cost of an increased axial resolution. In contrast, conventional US generally has an imaging frequency in the 2–15 MHz range and an axial resolution as fine as 200 µm^17,19^. The superior microUS resolution allows for visualization of prostate features and tumors that are not resolvable on conventional US which may facilitate tumor visualization (Fig. 1)^20^. While this manuscript focuses on microUS, readers should be aware that there are also ongoing efforts to develop alternative US techniques for prostate cancer visualization including contrast-enhanced ultrasound, quantitative ultrasound, elastography, and multi-parametric US^21–25^. Furthermore, these techniques can be applied to microUS imaging which can allow for further improvement in ultrasound imaging of prostate cancer as techniques are developed^22,26^.

MicroUS is a promising new modality, however it has not been robustly evaluated against mpMRI in clinical studies validated by ground truth histology. Previous studies validated with tracked biopsy have demonstrated similar cancer detection rates between microUS (35%) and mpMRI (39%) with comparable sensitivities (93% vs. 94%) and specificities (21% vs. 17%)^19,27,28^. Although these studies have demonstrated promise for microUS imaging, the gold standard evaluation for prostate imaging is validation against ground truth whole-mount (WM) pathology slides. Unfortunately, such a study is challenging because it is difficult to co-register imaging planes with pathology slides, because the former are acquired in a para-sagittal sweep and the latter are axial. Moreover, probe rotation is performed manually and thus prone to inconsistent tissue deformation and untracked imaging plane translations. Consequently, most studies comparing microUS and MRI have relied on cognitive registration of microUS and pathology through placement of an ROI into one of 12 corresponding template zones within the prostate^29,30^. Although this approach is sufficient for assessing general consensus between modalities, cognitive registration is limiting for the following reasons: (1) Zonal placement is subject to reviewer bias and expertise as correlation between non-orthogonal, and non-parallel microUS and WM image planes require significant cognitive effort and is prone to error (2) Registration error and overall quality of cognitive registration is not necessarily consistent across cases and reviewers, thus, specific ROI-lesion agreement metrics such as dice scores and overlap percentages cannot be accurately evaluated (3) Image masks created from cognitive registration are weak labels and can diminish the accuracy of predictive AI models for microUS, which would help facilitate widespread adoption of microUS.

Here, we demonstrate computational registration of microUS and MRI to WM pathology, with measurable registration accuracy and minimal bias, thus enabling rigorous assessment of both imaging modalities. In addition, this process facilitates generation of high quality, strongly labelled datasets which will pave the way for the development of deep learning models capable of automatically identifying cancerous lesions on microUS. We believe such models are a necessary prerequisite to widespread adoption of microUS as cognitive analysis of the images is challenging particularly in the biopsy setting where time is often limited. In earlier work, our group has validated an ex vivo microUS scanning setup and found comparable results between MRI and ex vivo microUS for prostate cancer identification through computational co-registration with WM pathology^20^. In this work, we build upon our approach with a validated, multi-step registration process for accurate co-registration of in vivo microUS and mpMRI with WM pathology. This approach is then utilized for quantitative analysis and comparison of microUS and mpMRI for blinded cancer detection in a clinical study with 15 patients. The goal of this study is two parts: (1) validate co-registration of microUS and MRI with ground truth WM pathology and (2) determine the diagnostic accuracy of microUS and mpMRI in a pilot clinical study.

## Methods

**Figure 2:**
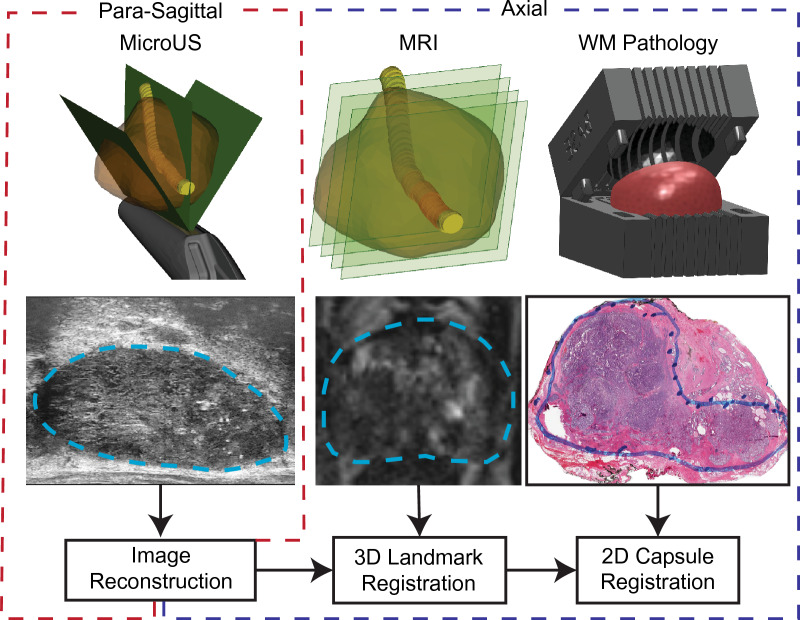
Overview of methodology of alignment of microUS images with MRI and WM pathology. MicroUS images are acquired in a para-sagittal fan sweep, while MRI is acquired in the axial plane. Following prostatectomy, the gland is sectioned in a pathology mold to align the WM sections with the MRI axial orientation. The para-sagittal microUS frames are reconstructed into a “true” sagittal orientation, then registered to MRI via a 3D landmark registration. Finally, MRI and microUS are registered to WM pathology via a 2D capsule registration.

A general overview of the three-step registration process is as follows (Fig. 2): After para-sagittal microUS images, axial MRI images, and axial WM pathology slides are obtained (1) The para-sagittal microUS images are reconstructed and interpolated to align with the MRI axial planes (2) The reconstructed microUS images are co-registered to the MRI via a 3D registration process based on common landmarks (3) The newly co-registered microUS and MRI datasets are registered to WM pathology via 2D capsule registration. The end result is a series of 3 transformation matrices that transform a point in para-sagittal microUS space to “true” sagittal microUS space, then to MRI space, and lastly to WM pathology space. Later in the pilot study, reviewers delineate regions suspicious for cancer on native microUS and MRI and the aforementioned matrices are retrospectively applied to register both datasets to ground truth WM pathology. All methods were carried out in accordance with the Declaration of Helsinki.

### Data collection

Fifteen patients set to undergo radical prostatectomy for cancer treatment gave informed consent and were enrolled into a registered clinical trial (NCT04299620) and UCLA Institutional Review Board approved study (IRB#19-001136). To be eligible for enrollment, each patient needed to have an mpMRI of their prostate with an associated UCLA radiologist report within a year prior to surgery, biopsy confirmed prostate cancer, and no prior treatment. Any radiologist identified PI-RADSv2.1 (Prostate Imaging Reporting and Data System) score 3 or higher suspicious regions were exported from the mpMRI and stored.

In the operating room on the day of surgery, each patient received at least 3 transrectal microUS sweeps of their prostate at large, medium, and small imaging presets prior to the procedure. All microUS scans were performed by the same experienced urologist to ensure consistency across patients. Immediately after a scan was obtained it was reviewed and, if necessary, repeated to confirm sufficient quality and that the entire prostate was captured. After prostatectomy the prostate was placed in a patient-specific 3D printed histology alignment mold and sectioned in accordance with routine clinical practice at UCLA^31^. These molds are specifically designed to section the prostate in the axial orientation to allow for direct comparison between MRI and final pathology. Using a microtome, WM pathology slides were made from each section and subsequently scanned at 20X magnification and saved digitally. Cancerous lesions were delineated on the WM slides by an experienced genitourinary pathologist. In summary, an axial mpMRI scan, a para-sagittal microUS sweep, and axial WM pathology slides were acquired from 15 patients (Fig. 2).

### MicroUS image plane reconstruction

**Figure 3:**
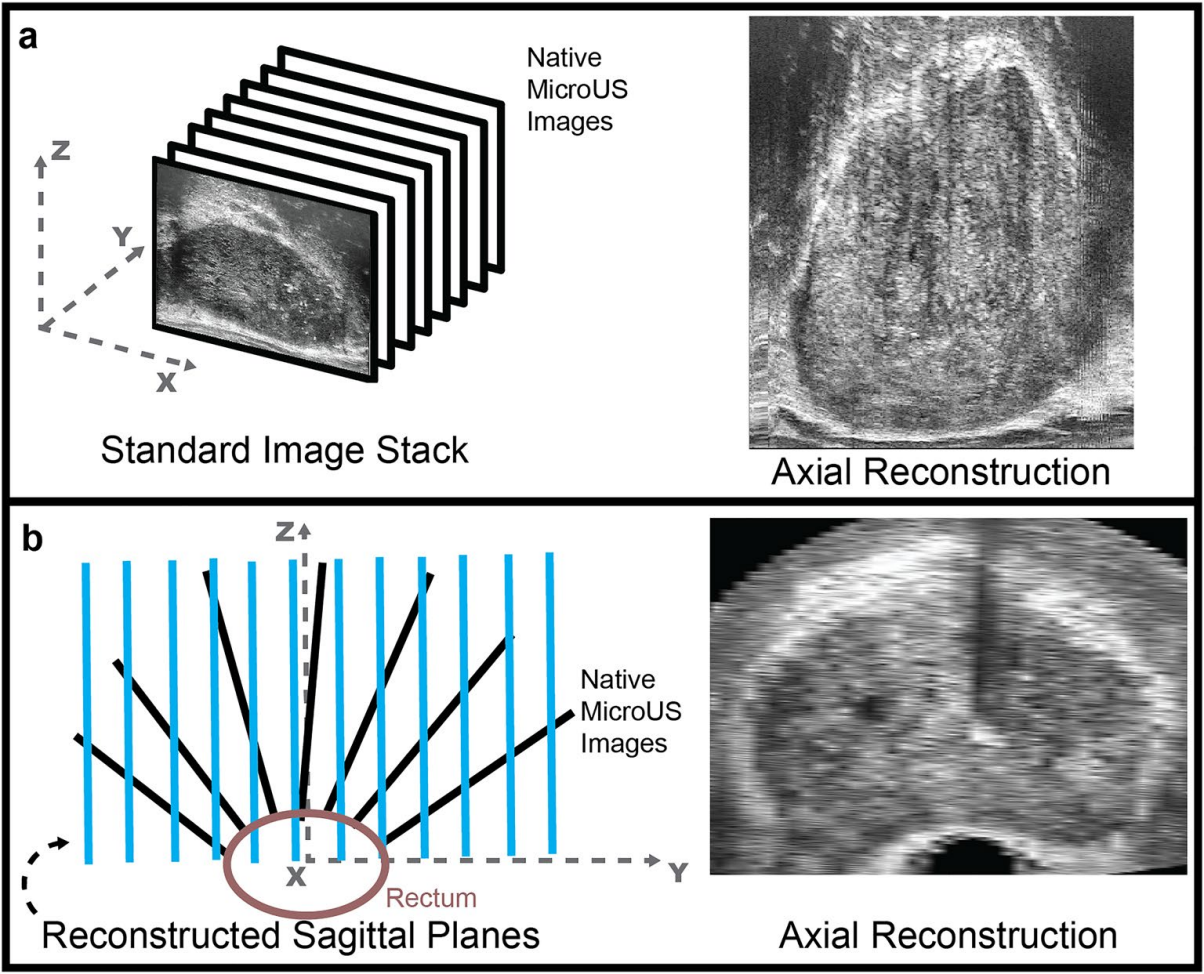
(**a**) Loading consecutive images into a DICOM viewer without accounting for scan angle results in a warped prostate shape that is not representative of the actual gland. (**b**) By accounting for the scan angle and imaging offset it is possible to reconstruct sagittal images (blue) that, when loaded into a DICOM viewer, preserve the geometric shape of the gland to facilitate axial viewing and 3D reconstruction.

The first step to register the para-sagittal microUS sweep with the axial MRI and WM datasets required the microUS image data to be reconstructed into parallel frames. Since every frame in an imaging sweep was acquired at a different angle in a para-sagittal sweep, the data cannot be loaded into a standard DICOM viewer without losing geometric positioning of the frames (Fig. 3a). A custom MATLAB (MathWorks) script was used to reconstruct the microUS images in the sagittal orientation through positioning in 3D space, resampling, and interpolation. The microUS images were obtained as a 300-frame sweep from the right to left side of the prostate with each image at a varying angle of transducer rotation. The native image frames can be thought of as 2D planes in a cylindrical coordinate system rotated around the rectum (x-axis) at a specific angle and offset from the center of rotation. To mimic this, a rotation around the x-axis was applied to the pixel coordinates of each frame using the stored angle metadata. Due to the microUS probe design, there is a 2 mm offset from the edge of the US transducer and the first row of pixels acquired in the image. To account for this, each image was shifted above the x-axis by 2 mm prior to rotation. After rotating all the images, empty parallel planes with 1 mm spacing along the y-axis (sagittal orientation) were populated with any image data that lies within y ± 0.5 mm of the plane. A gridded interpolation step was performed to fill any holes in the new image planes. These new images were then saved as a DICOM series with embedded pixel spacing and coordinate locations. When loaded in a standard DICOM viewer, these reconstructed scans allow for correct visualization of the prostate in traditional orientations (axial, sagittal, and coronal) (Fig. 3b).

### MicroUS to MRI registration

#### Registration process

Following microUS image plane reconstruction, the next step is to account for tissue deformation and any misalignment from the reconstruction step. During microUS interrogation of the prostate, the probe deforms the prostate causing compression of the gland in-plane. Furthermore, only the roll (rotation around the x-axis) of the US probe was tracked and recorded during scanning, so any pitch, yaw, or translation has not been accounted for at this stage. The reconstruction process described in the prior step is designed to approximate the shape of the prostate, but a corrective registration step is required. To account for this, a landmark registration with MRI was performed. MRI was chosen over direct registration to WM pathology due to the superior sampling density of MRI and to avoid potentially confounding factors for tissue deformation from WM slide shrinkage during fixation and processing. Additionally, WM pathology is generally limited to at most 6–7 slides per case with approximately 4.5 mm spacing between slides, whereas the majority of MRI data was acquired with 1.5 mm spacing providing 3 times the sampling density registration.

**Figure 4:**
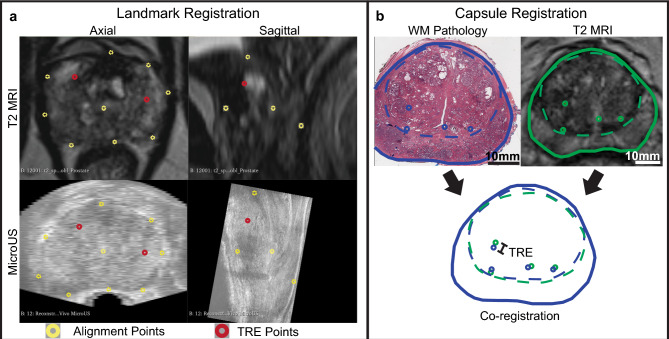
(**a**) Landmark registration between microUS and MRI. Points along the urethra and the capsule were selected to inform the TPS transformation (yellow). The distance between five internal anatomical fiducials (red) were used to evaluate TRE. (**b**) Capsule registration between MRI and WM pathology. The distance between fiducials (circles) were used to evaluate TRE and the overlap of the transition zone (dashed lines) was used to determine DICE scores.

For microUS to MRI registration, the reconstructed microUS and MR images were loaded into 3D Slicer^32^ and the microUS sweep was visually aligned to the MRI via translation and rotation. Following visual alignment, a landmark based thin-plate spline (TPS) registration was performed. Landmark selection was limited to selection of 3 common points along the urethra at the apex, mid-gland, and base, as well as points along the capsule in 3 axial frames in the apex, mid-gland, and base (Fig. 4a).

#### Registration accuracy assessment

The accuracy of the registration was then assessed by selecting 5 internal landmarks (excluding the urethra) and measuring the distance between them to get the target registration error (TRE). Landmarks were generally distributed throughout the prostate rather than clustered in the same location (e.g., apex, base, mid-gland, left, right).

### MRI to WM pathology registration

#### Registration process

After microUS registration with MRI, a second registration step is performed to register both microUS and MRI with WM pathology. Before this can be done, the WM slides must be matched to a specific MRI image. The general location and the order of each histology slide is known. However, since the thickness of each histology section is 4.5 mm and the MRI thickness in our dataset is usually 1.5 mm, there are at least 3 possible MRI images that could correspond to a given WM slide. To address this, an experienced reviewer matched each WM slide to a corresponding MRI slide using the knowledge of the slide location, size and shape of the capsule on MRI and WM, anatomical features (e.g., urethra, seminal vesicles, BPH nodules, and calcifications), and the relative spacing from other MRI matched WM slides. After matching all slides, it was confirmed that exactly one WM slide was contained within every 3 MRI frames (when MRI slice thickness was 1.5 mm). Following this process, WM slides are able to be matched with an error of approximately 1.5 mm (or one MRI frame) as reported by an earlier study from our group^33^.

Using MRI and WM capsule segmentations, the MRI was co-registered to the WM slides through a custom MATLAB script. The registration involved a rigid transformation to account for scaling and in-plane rotational misalignment, followed by a non-rigid TPS transformation using control points on the prostate capsule to account for specimen deformation as performed in earlier studies^34,35^.

#### Registration accuracy assessment

In order to assess registration accuracy, matched image data were assessed by four reviewers (3 urologists, 1 imaging scientist). Using a DICOM viewer (Weasis Medical Viewer), each of the 15 subjects was reviewed by at least two reviewers and each reviewer was randomly assigned a subset of eight cases to review. For each case, reviewers were asked to mark up to four common anatomical landmarks as well as outline the transition zone and capsule on both MRI and WM for each matched image pair (Fig. 4b). The MRI was then co-registered to WM for each case and each reviewer. The distance in millimeters between identified landmarks was recorded as the TRE, and the dice similarity coefficient was calculated from the overlap of the identified transition zones.

### Pilot clinical study

#### Whole mount pathology

As described earlier, in accordance with normal clinical practice, an experienced genitourinary pathologist reviewed the WM pathology slides and identified the prostate cancer boundaries as well as the International Society of Urological Pathology (ISUP) grade group of the tumors.

#### MRI

For MRI, any delineated PI-RADSv2.1 target from the original mpMRI prior to surgery was imported. In addition, using the mpMRI scan, an experienced radiologist with over ten years of experience with PI-RADS was asked to delineate any suspicious regions on T2 MR images with the knowledge the patient received a prostatectomy for prostate cancer (retrospective MRI). This was necessary because the PI-RADSv2.1 protocol prioritizes specificity and identification of the center of the index lesion as a triage for biopsy. Consequently, this methodology underestimates tumor extent in an effort to minimize unnecessary biopsy procedures^14^. However, we are interested in analyzing the ability of microUS and MRI to identify the margins of prostate cancer, which is critical for treatment decisions and focal therapy planning. Furthermore, given microUS is a relatively new imaging modality, there are limited numbers of readers familiar with the technology and even fewer expert readers. As a result, the experienced readers available for this study had knowledge of the study design and were aware it is validated via WM pathology, which is only available for prostatectomy patients. For the above reasons, both the MRI and microUS reviewers were aware the patient had a prostatectomy prior to review, but were blinded to the WM pathology and other clinical history of the patient (e.g., cancer grade, PSA, biopsy results).

#### MicroUS

For microUS, the same four reviewers in the landmark study reviewed the native microUS sweeps and were asked to identify up to two suspicious lesions per subject to ensure only the most suspicious regions were annotated. Reviewers had varying levels of microUS experience but they all completed an online training module from the microUS manufacturer to ensure baseline competency. However, the clinical experience of each reviewer varied (number of microUS biopsies performed or observed). Reviewers 1–3 had performed or observed over 50 microUS targeted biopsies at the time of review and frequently interpret microUS scans, while reviewer 4 had knowledge of the system but had not attempted interpretation outside of the training module. Similar to the landmark study, each subject was reviewed by at least two reviewers with each reviewer assigned to the opposite subset of cases they reviewed in the prior landmark study to avoid any previous knowledge bias. As an added precaution, there was also a minimum 90-day washout period between reviews to avoid bias. Reviewers were blinded to all clinical parameters but were aware the patient had a prostatectomy for prostate cancer, similar to prior final histology based studies for microUS^29^.

#### Registration

**Figure 5:**
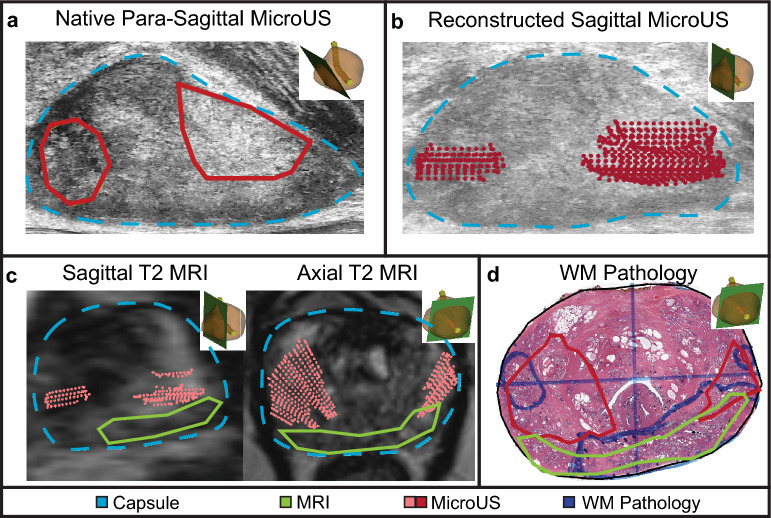
Complete registration process from microUS to WM pathology. (**a**) MicroUS reviewer delineations (red) are made on native microUS imaging. (**b**) These markups are turned into a point-cloud and positioned in 3D space similar to the image reconstruction. (**c**) The transformation matrices for microUS to MRI registration are applied to the markup. (**d**) Lastly, the microUS and MRI (green) delineations are co-registered with WM pathology.

Following the described registration process, the microUS annotations (Fig. 5a) were first transformed into a point-cloud and oriented in 3D space in the same manner as the reconstruction of the microUS images (Fig. 5b). The annotations were then registered to MRI via the stored transformation matrices created from the 3D landmark registration between microUS and MRI (Fig. 5c). After registering microUS annotations with MRI, both the MRI and microUS annotations were registered to the WM pathology following the rigid and non-rigid MRI to WM capsule registration (Fig. 5d). Using density-based spatial clustering of applications with noise (DBSCAN), the microUS point-clouds were transformed into outlined regions-of-interest (ROIs). For each review of the 15 cases, pixel level detection metrics were quantified, and figures were created for each of the WM slides overlaid with the annotations from prospective PI-RADSv2.1 MRI (pMRI), retrospective MRI (rMRI), and microUS reads.

## Results

### Data collection

Each surgery was performed by one of two high volume surgeons using a robotic assisted laparoscopic prostatectomy approach. The median patient age was 63 [58–68] (median [IQR]). Median preoperative PSA was 8.8 [6.3–11.3]. The final pathology revealed 5 patients with organ confined disease (T2), 7 with extra prostatic extension (T3a), and 3 with seminal vesicle invasion (T3b). There was a total of 19 significant lesions in the patient population. The median ISUP Grade Group was assigned to each lesion. The average number of lesions per prostate was 1.6 ± 0.6 (mean ± standard deviation). For the index lesion (highest grade and/or largest tumor) the average largest axial dimension was 29.4 ± 11.9 mm with the following ISUP grade group (GG) distribution: GG2: 8, GG3: 5, GG4: 1, and GG5: 1. There were 4 significant secondary lesions, all of which were GG2 and 5 clinically insignificant GG1 secondary lesions.

### MicroUS image plane reconstruction

**Figure 6:**
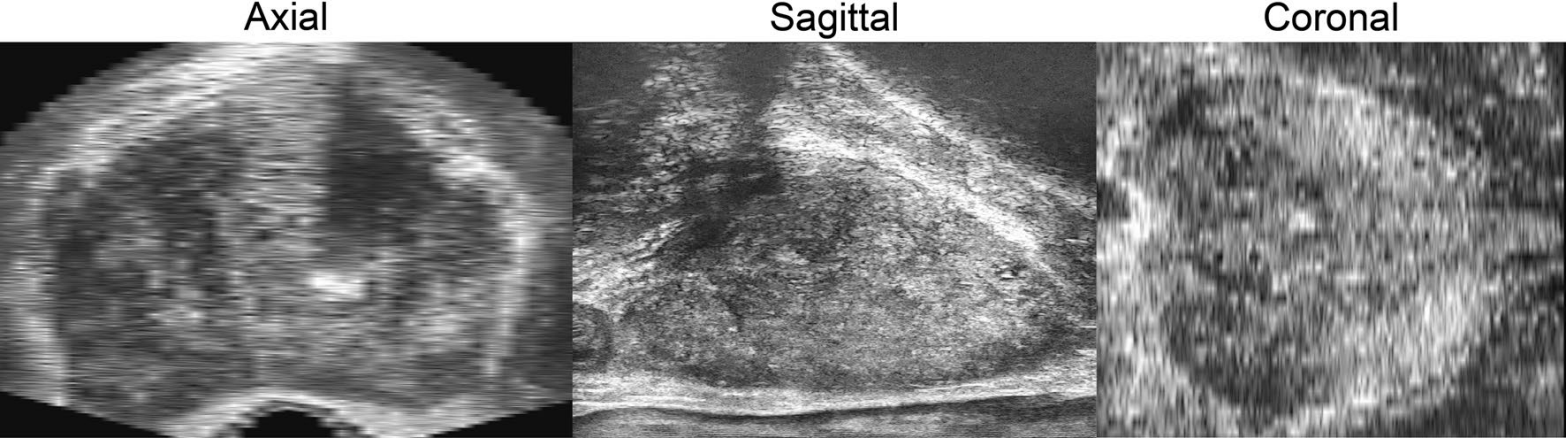
Example of a set of microUS images reconstructed in axial, sagittal, and coronal orientations. Sagittal images were reconstructed via a MATLAB script allowing for visualization of the axial and coronal planes in a DICOM viewer. Sagittal images are of sufficient quality to enable accurate visualization of anatomical features.

A total of 26 different microUS scans were reconstructed (1–2 per patient) with a slice thickness of 1 mm. On average the reconstructed scan contained 68 ± 8 (mean ± standard deviation) images created from the original 300 frame scan. The reconstructed images are saved as DICOM files and contain embedded pixel spacing, slice position, and slice thickness information allowing for clear visualization of the prostate in the axial sagittal and coronal planes in any standard DICOM viewer (Fig. 6). Images correctly represented the shape of the prostate while preserving image quality for visualization of anatomical features.

### MicroUS to MRI registration

**Figure 7:**
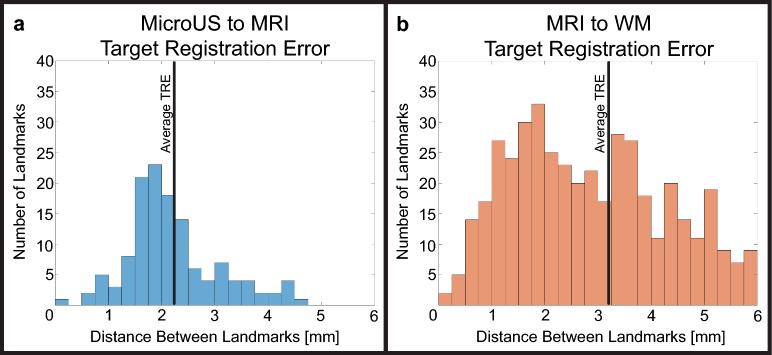
Distance between anatomical landmarks following microUS to MRI (**a**), and MRI to WM pathology registration (**b**).

Following the registration of the reconstructed microUS scan with T2 MRI, five common anatomical landmarks (excluding the urethra) were selected from varying regions of the prostate for each of the 26 scans. A total of 130 landmarks were identified and the distance between them measured. The resulting average TRE was 2.23 ± 0.08 mm (mean ± standard error) (Fig. 7a).

### MRI to WM pathology registration

MRI was co-registered to WM pathology on a subset of 8 patients for each of the 4 reviewers, for a total of 32 registrations comprised of 159 slides and 472 TRE landmarks. Landmarks that were greater than 2 standard deviations outside the mean TRE were removed as they were determined to be a result of incorrect landmark selection. The average TRE between landmarks on MRI and WM was 3.20 ± 0.08 mm (mean ± standard error) (Fig. 7b). In total only 26 landmarks from an original 498 (5%) were excluded. The average dice similarity coefficient of the transition zone was 0.84 ± 0.01 (mean ± standard error). When comparing reviewers, landmarks were not excluded in order to preserve reviewer variability. There was no significant difference between reviewers for TRE (*p* = 0.15) and transition zone dice scores (*p* = 0.62, Kruskal–Wallis). The combined TRE (summation in quadrature) of microUS to WM pathology from the 3 registration steps was 3.90 ± 0.11 mm.

### Pilot clinical study

**Figure 8:**
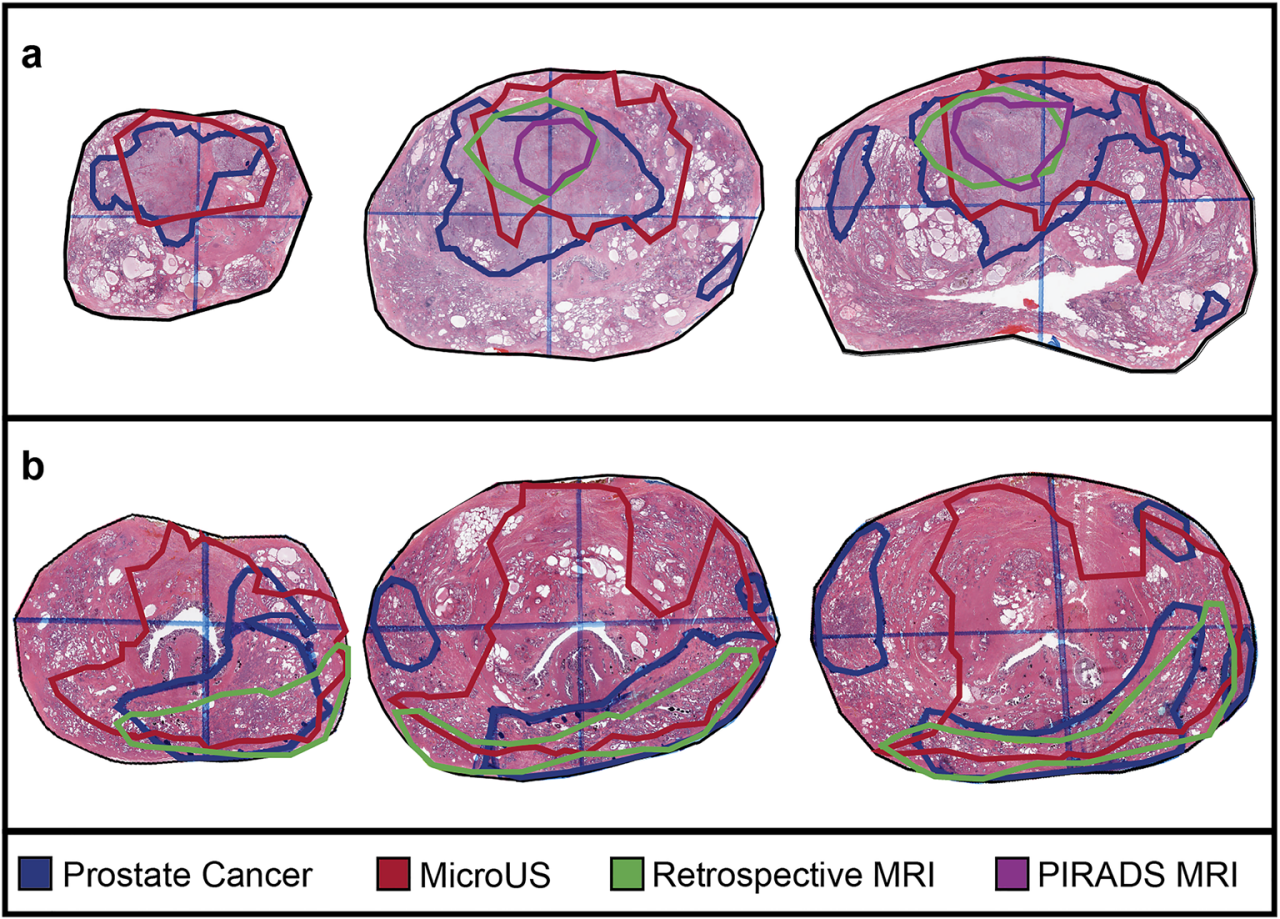
Example co-registration outputs of microUS and MRI with WM pathology for two cases (**a** and **b**).

A total of 32 microUS reviews (8 per reviewer) were registered to the associated MRI and subsequently registered to WM pathology. Two examples of registration of microUS and MRI annotations to WM pathology are shown below (Fig. 8). Only clinically significant, grade group 2 or greater, lesions were considered for this analysis. Results are reported as mean ± standard error where appropriate.

**Figure 9:**
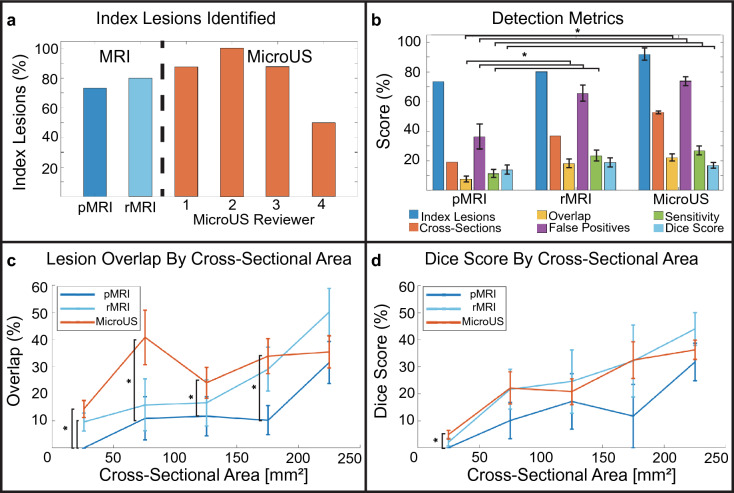
Assessment of microUS and MRI against WM pathology in a 15-patient clinical study. (**a**) Index lesions identified by modality and reviewer. (**b**) Combined reviewer detection metrics for each modality at the patient level (index lesions), cross-section level (cross-sections), and pixel level (overlap, false positives, sensitivity, dice score). Lesion overlap (**c**) and dice score (**d**) for each modality stratified into groups by lesion cross-sectional area. (* indicates *p* < 0.05, Wilcoxon rank sum. Error bars are ± one standard error).

Due to the rigorous nature of the registration process, analysis can be performed at increasing levels of granularity starting at the patient level, then the cross-section level and finally the pixel level. At the patient level, a total of 11/15 (73.3%) index lesions were identified by pMRI and 12/15 (80%) were identified by rMRI. For each individual microUS reviewer: 7/8 (88%), 8/8 (100%), 7/8 (88%), and 4/8 (50%) index lesions were identified respectively (Fig. 9a). In a combined microUS analysis, where reviewer 4 was removed, 91.7 ± 4.2% of index lesions were identified (Fig. 9b). MRI did not detect any index lesions that were not detected by microUS. If any part of an ROI overlapped the index lesion it was considered a correct identification. Reviewer 4 was removed from combined microUS analysis due to reduced expertise and poor index lesion identification. Reviewers 1–3 (2 attending urologists and 1 expert imaging scientist) had performed and observed over 50 microUS biopsies at the time of review, whereas reviewer 4 (urologic fellow) had not performed any microUS biopsies at the time of review. Furthermore, there was a significant decrease between reviewer 4 and the other 3 reviewers (*p* < 0.0001, Kruskal–Wallis) when analyzing ROI-tumor overlap.

At the cross-section level, a total of 90 clinically significant lesions cross-Sections (2D slice of a lesion on WM pathology) were present across the 82 WM slides from all 15 patients. The average ± standard error and median [IQR] area of these lesion cross-sections was 89.0 ± 11.7 mm^2^ and 37 mm^2^ [1–130] respectively. Of these 90 lesion cross-sections, pMRI identified 17/90 (18.9%) whereas rMRI exhibited improved performance with 33/90 (36.7%). MicroUS had even better performance identifying 85/162 (52.5 ± 0.1%) lesion cross-sections between the three reviewers (Fig. 9b). Similar to the index lesion analysis, if any ROI overlapped a lesion cross-section it was considered a correct identification. Identification of lesion cross-sections provides a metric to assess the ability of the imaging modalities to identify tumor extent.

The following quantitative metrics were analyzed at the pixel level for increased granularity and improved margin evaluation which is crucial for focal therapy planning. For example, the overlap between an ROI and a cancerous lesion was decided for every pixel contained within a lesion or ROI.

The lesion overlap was defined as equation (1):

1 $$Overlap \left( \% \right) = \left( {\frac{Lesion Area \cap ROI Area}{{Lesion Area}}} \right)100$$

The overlap percentage accounts for the amount of a lesion cross-section encapsulated by a modality. The average overlap for pMRI, rMRI, and microUS were 7.4 ± 1.9%, 17.1 ± 2.8% and 22.8 ± 2.4% respectively (Fig. 9b). Interestingly, there was a significant difference between both microUS and pMRI, and rMRI and pMRI (*p* < 0.001, Wilcoxon rank sum) whereas microUS and rMRI exhibited no statistical difference. This suggest that both rMRI and microUS identified significantly more tumor extent than pMRI. The reduction in encapsulation between pMRI and rMRI can likely be attributed to the primary focus of PI-RADSv2.1 on identifying the core of the index lesion while preserving specificity as part of the decision to refer a patient to biopsy. The overlap percentage of each modality was also stratified by cross-sectional area to show how tumor size can affect diagnostic ability (Fig. 9c). As expected, overlap generally improved as lesion size increased with pMRI, rMRI, and microUS increasing from 0 ± 0%, 8.9 ± 3.2%, and 15.2 ± 3.1% to 31.5 ± 7.8%, 50.1 ± 8.8%, and 35.5 ± 5.9% respectively. There was also a significant difference between microUS and pMRI for all but the largest groups of lesions (*p *< 0.05, Wilcoxon rank sum) indicating that microUS may outperform pMRI for tumor extent when detecting all but the largest lesions. However, given that there was no statistical difference between microUS and rMRI, it is likely that the deficiencies of pMRI are not due to limitations of the MRI modality but rather the PI-RADSv2.1 protocol which is not designed for optimizing overlap.

The false positive metric was defined as Eq. (2):

2 $$False{ }\;Positive{ }\left( {\text{\% }} \right) = \left( {1 - \frac{{Lesion{ }\;Area{ } \cap { }ROI{ }\;Area}}{{ROI{ }\;Area}}} \right){*}100$$

This metric accounts for the amount of an ROI that does not overlap a lesion and is analogous to the specificity of a modality. False positive percentages were reported instead of specificity for the following reasons: (1) To avoid potentially misleading values which arise as the pixel level granularity of the analysis results in inflated specificity values (approximately 95% for all modalities) when compared to conventional zonal based studies^19,30^ (2) To allow for more opportunities for one imaging modality to distinguish itself from the other since the area of the ROI is always substantially smaller than the benign (negative) area. At 36.2 ± 8.6%, the pMRI false positive ratio was significantly better than rMRI (65.4 ± 5.5%) and microUS (73.7 ± 3.0%) (*p *< 0.01, Wilcoxon rank sum). This reflects the prioritization of specificity in the PI-RADSv2.1 protocol. Furthermore, only 5/22 (22.7%) of pMRI annotations completely missed a significant lesion cross-section, whereas rMRI and microUS had 20/49 (40.8%) and 41/107(38.3%) respectively. Collectively, this indicates pMRI has superior specificity to both rMRI and microUS. Interestingly, microUS and rMRI had similar rates of both false positives and completely false ROIs.

Dice scores evaluate the similarity between two ROIs, defined as Eq. (3):

3 $$DICE = 2\left( {\frac{{Lesion{ }\;Area{ } \cap { }ROI{ }\;Area}}{{ROI{ }\;Area + Lesion{ }\;Area}}} \right){*}100$$

Since this metric accounts for both false positive regions and false negative regions, it is a single metric that represents both sensitivity and specificity. For pMRI, rMRI, and microUS the dice scores were 13.9 ± 3.1%, 18.7 ± 3.1%, and 16.6 ± 1.9% respectively (Fig. 9b). There was a significant difference between microUS and pMRI dice scores (*p* < 0.05, Wilcoxon rank sum) indicating microUS allows for better similarity between reviewer identified ROIs and true tumor boundaries than pMRI. Similar to overlap percentage, dice scores were stratified based on cross-sectional area to analyze how tumor size affects diagnostic accuracy. As expected, dice scores generally improved as lesion size increased with pMRI, rMRI, and microUS increasing from 0 ± 0%, 1.9 ± 1.3%, and 4.6 ± 1.5% to 31.7 ± 7.0%, 44.0 ± 6.1%, and 36.3 ± 3.6% respectively (Fig. 9d).

The sensitivity of each modality was defined as Eq. (4):

4 $$Sensitivity = { }\left( {\frac{{Lesion{ }\;Area_{Slide} { } \cap { }ROI{ }\;Area_{Slide} }}{{Lesion{ }\;Area_{Slide} }}} \right){*}100$$

Although similar to the lesion overlap percentage, sensitivity was calculated per slide rather than per lesion. As such, slides with multiple lesion foci were grouped together. For pMRI, rMRI, and microUS the sensitivity was 11.1 ± 2.7, 22.1 ± 3.6%, and 26.7 ± 3.0% respectively (Fig. 9b). A significant difference was found between pMRI and rMRI, and pMRI and microUS (*p *< 0.01, Wilcoxon rank sum) further indicating the improved ability of microUS and rMRI over pMRI for assessing tumor extent.

## Discussion

Conventional US is non-diagnostic for prostate cancer, as such mpMRI is the recommended imaging modality for prostate cancer detection^6,11,12^. However, mpMRI is resource intensive, limiting its use to large centers. High-resolution microUS has been introduced as a potential low-cost alternative with promising preliminary results. Prior studies have relied on biopsies or cognitive zonal registration with histology to compare the diagnostic accuracy of microUS and MRI^29,30^. Computational registration of microUS and MRI with ground truth WM pathology facilitates more rigorous comparison of the two imaging modalities, however, to date that has not been demonstrated as the native orientation of microUS imaging makes direct registration with WM pathology difficult.

In this work we developed and validated the first multi-step process for co-registration of microUS, MRI, and WM pathology, and subsequently assessed the ability of expert microUS and MRI reviewers to identify prostate cancer against ground truth WM pathology. In doing so, we have created a validated methodology that allows for rigorous comparison of microUS and MRI, as well as an approach for labeling microUS images with ground truth histology for predictive model training. The three-step process consists of (1) microUS para-sagittal image reconstruction into the true sagittal orientation, (2) 3D alignment and TPS registration of the reconstructed microUS images with MRI, and (3) 2D alignment and TPS registration of MRI with WM pathology slides.

The first step, microUS image reconstruction, approximates the shape of the prostate capsule at the time of the US scan. This reduces the magnitude of subsequent registration steps, reducing opportunities for misregistration. We have demonstrated it is feasible to reconstruct sagittal microUS images from native images acquired from a handheld transrectal, para-sagittal sweep of the prostate. The image quality from these reconstructions is adequate to identify intraprostatic fiducials such as the urethra, and preserves the anatomical prostate shape. Furthermore, since these images are used as an intermediary in the registration process, reviewers are able to delineate the native microUS images without any loss of image quality. This is an improvement on our prior work which required reviewers to delineate ex vivo imaging in order to align with MRI and WM pathology, which is not necessarily representative of the in vivo imaging environment^20^.

In the second step, reconstructed microUS images are co-registered to MRI using a landmark TPS registration. This accounts for tissue deformation during microUS scanning and possible reconstruction error from limited probe position tracking. Intermediate registration to MRI was chosen over direct registration to WM pathology for the following reasons: (1) MRI has superior through plane resolution compared to WM (1.5 mm vs. 4.5 mm) providing more information for registration (2) MRI is in vivo and more representative of the microUS imaging environment than WM slides, and (3) WM pathology processing causes the tissue to shrink introducing additional sources of error^36^. The measured TRE for microUS to MRI co-registration was 2.23 ± 0.08 mm which is within the 2–3 mm TRE for commercial MRI-US fusion systems^37,38^.

The third and final step, 2D MRI to WM capsule registration, allows for direct comparison with ground truth histology. Since microUS is fused to MRI in step two, the MRI to WM transformation matrix can be applied to both MRI and microUS annotations. The average TRE for MRI to WM was 3.20 ± 0.08 within the range reported in literature of 1–5 mm^39^ resulting in a total registration error of 3.90 ± 0.11 mm for microUS to WM pathology. The registration accuracy is further validated by the pilot clinical study, where MRI and microUS identified 73.3–91.7% of the index lesions, consistent with earlier studies^7–9,29,30,40^.

After validating each step of the registration process, it was applied to 15 patients. Each patient had a pre-operative MRI read, a retrospective MRI read, and at least two independent microUS reads registered to final pathology.

The microUS results emphasize the crucial role of reviewer training and experience beyond baseline competency. Notably, reviewer 4, being the least experienced, achieved significantly inferior results compared to the other reviewers (*p* < 0.0001). This also highlights a potential value of future deep learning models to alleviate both inter-reviewer variability and the need for extensive training.

In the pilot clinical study, microUS demonstrated non-inferiority to both pMRI and rMRI for identifying prostate cancer index lesions and tumor extent. Specifically, microUS identified a higher percentage of index lesions (91.7 ± 4.2%) compared to pMRI (73.3%) and rMRI (80%). It is vital for an imaging modality to identify the index lesion as it often drives treatment decisions. MicroUS also identified a higher percentage of lesion cross-sections with better overlap (52.5 ± 0.1% and 22.8 ± 2.4%) than both pMRI (18.9% and 7.4 ± 1.9%) and rMRI (36.7% and 17.1 ± 2.8%). Lesion cross-sections and overlap percentages are representative of tumor extent throughout the prostate and are related to a modality’s ability to identify tumor margins. Patient treatment decisions can be affected by cancer volume and location in the prostate. As such, margin identification is important for cancer staging and deciding treatment options, especially for focal therapy where only part of the gland is treated.

MicroUS also performed comparably to rMRI for both sensitivity (26.7  ± 3.0% vs 22.1 ± 3.6%) and dice scores (16.6 ± 1.9% vs 18.7 ± 3.1%), with both outperforming pMRI (11.1 ± 2.7% and 13.9 ± 3.1%). Conversely, MicroUS and rMRI had similarly high false positive percentages (73.7 ± 3.0% and 65.4 ± 5.5% respectively) and completely false ROIs (38.3% and 40.8% respectively) compared to pMRI (36.2 ± 8.6% and 22.7%). This emphasizes that PI-RADSv2.1 is designed to prioritize specificity of MRI at the cost of sensitivity to avoid unnecessary biopsies. Perhaps the specificity of microUS, often noted to be worse than pMRI ^27,41,42^ can be increased through the refinement of the microUS diagnosis protocol (PRI-MUS) similar to improved iterations of PI-RADS raising the specificity of MRI^7^.

It is worth noting that the recorded results for sensitivity, dice scores, and overlap percentage are lower and the false positive percentages are higher than reported in other related studies^19,27,29,30^. This can be attributed to the granularity of the analysis performed. For these metrics each pixel was treated as a possible true positive, false positive, true negative, or false negative event. Any misalignment between regions reduces the modalities accuracy allowing for more opportunities for one modality to outperform the other, which is important for this study given the limited sample size (N = 15). Other studies in the space often rely on zonal based analysis, analogous to the study by Turkbey et al.^43^, where the quantity of possible true positive, false positive, true negative, and false negative events are limited to twelve zones. In general, with increased granularity, the overlap, sensitivity, and dice scores decrease and false positive ratios increase. An example of this can be seen in similar work co-registering WM to MRI with a pixel spacing of ~ 0.6 mm, which reported MRI sensitivity and specificity values of 37% and 97.9% respectively, whereas traditional zonal MRI studies report sensitivities and specificities of 86–92% and 60–83% respectively^7–9,44^. To avoid reporting potentially misleading values, specificity values of each modality were intentionally not reported, and instead the false positive ratio was used as a surrogate for specificity. With increased granularity, the specificity for all modalities and all reviewers was approximately 95% since it is related to the number of true negative events, and the majority of WM slides were comprised of mainly benign tissue. Although the specific results from this study are not directly comparable to other zonal based studies, the general trends can be compared.

The results presented in this study provide good agreement with the larger body of literature in the field. Specifically, microUS demonstrated non-inferiority compared with MRI for index lesion identification and detection of tumor extent^19,27,29,30^. This is particularly important given the reduced cost of microUS compared to mpMRI. However, microUS had higher false positive ratios than MRI, similar to other studies^27,41,42^. Additionally, this study demonstrated reviewer expertise is vital for identification of prostate cancer on microUS; a possible reason for earlier studies having lower detection rates compared to later studies^19,27,45^.

This study also highlights a potentially synergistic relationship with mpMRI and microUS, wherein, MRI is used to triage patients for biopsy and identify the index lesion and microUS is used during biopsy to identify tumor extent throughout the prostate and better inform tumor margins for treatment decisions. MRI is sensitive for index lesion cancer and maintains high specificity, making it an excellent system for triaging patients for biopsy. However, PI-RADSv2.1 severely underestimates the extent of the lesion in patients as reported in this work and in earlier studies^14^. While microUS has reduced specificity, this generally would only result in extra biopsy cores rather than performing a biopsy on a patient who does not need it. Whereas the improved detection of tumor extent under microUS could result in better tumor margin identification. This is particularly valuable in an era where focal therapy modalities have been developed with the ability to create precise ablation zones and are thus capable of achieving oncologic control if tumor margins are evident e.g. focal HIFU^46^ or laser ablation with real-time monitoring^47,48^.

This study also demonstrated retrospective MRI reads with knowledge of existing cancer, greatly improved identification of tumor extent over a standard PI-RADSv2.1 read, while conversely lowering the overall specificity for prostate cancer. This suggests tumor margin identification could be improved by re-reading MRI scans after cancer is confirmed via biopsy.

There are a few limitations of this study. The registration process presented here is accurate, however, it is not fully automated, requiring WM to MRI matching and landmark selection for microUS to MRI registration. This can potentially be improved upon with the implementation of algorithms for MRI to WM matching as well as capsule and urethra identification. Another potential limitation of the registration process, inherent to side-fire transrectal US, is larger prostates will have more signal attenuation and larger distances between consecutive images in the anterior regions. This could diminish reconstruction quality and registration. However, this may be a minimal factor as the largest prostate in this cohort was 108 g (20 g is average^49^) and the average microUS to MRI registration error for this patient was only 0.16 mm higher than the total average. Moreover, both microUS reviewers correctly identified all significant lesions for this patient. Additionally, the diagnostic metrics derived here should be interpreted with some caveats. Firstly, the limited sample size (N = 15 patients) reduces statistical power and also indicates that a broad of spectrum of tumor presentations (size, location, ISUP grade group) was not examined. Secondly, the patient cohort consists of only prostatectomy patients, an inherent limitation of WM pathology-based studies. As such, the patient population likely has more aggressive disease than an equivalent biopsy or focal therapy cohort. Additionally, due to the relatively limited number of WM pathology slides per prostate and the differences between identifying an ROI and taking an actual biopsy, a correct detection of cancer on microUS or MRI could still lead to a negative targeted biopsy core, particularly for transrectal biopsies. For this reason, we avoided approximating correct biopsy targets since a core could have been taken from a region of an ROI that did not overlap cancer, especially when accounting for tissue deformation and needle deflection during sampling. Moreover, microUS reviewers were aware the patient had a prostatectomy for prostate cancer due to limited availability of expert microUS readers and their knowledge of the study being validated by WM pathology. As mentioned earlier, this limitation is present in earlier final pathology based studies reported in literature^29^. To account for this limitation, the MRI was also reviewed retrospectively with the same knowledge allowing for an equal comparison between microUS and MRI. It is also worth noting that the microUS images were effectively recorded scans, subsequently the microUS reviewers were not able to utilize potential scanning benefits from live US imaging such as variable compression and probe translation to further interrogate suspicious tissue. It also may not be indicative of a live interpretation in regards to time constraints during an actual biopsy. Furthermore, for non-expert reviewers, reading microUS images is challenging, evident from the performance of the excluded microUS reviewer. This emphasizes a need for automated predictive models for cancer detection to reduce the learning curve and cognitive load on physicians when interpreting microUS images during biopsies.

In contrast to MRI^50^, microUS-based AI models for automated tumor detection are much earlier in development and typically rely on weakly labeled biopsy data^51–53^. This is largely due to the absence of accurately labeled datasets which have been challenging to compile because of the complicated registration process. However, the approach that we have presented here greatly alleviates this problem and thus has the potential to spark the development of the requisite AI models that would greatly ease the adoption of microUS.

The registration process presented here has great utility in further characterizing the diagnostic capability of microUS. Interest in this area is rapidly growing as evidenced by recent publications^19,27,29,30,41,42^. In line with these studies, the pilot study that we have presented demonstrates that microUS has similar performance to MRI for identifying prostate cancer. This growing body of evidence suggests that microUS has the potential to be an inexpensive alternative or adjunct to MRI for prostate cancer visualization.

## Conclusion

MicroUS has the potential to greatly reduce the cost of imaging prostate cancer relative to MRI. However, direct registration with ground-truth WM pathology is challenging. To overcome this, we developed and validated the first methodology for accurate co-registration of microUS and MRI with WM pathology. The methodology was then successfully implemented in a multi-reviewer pilot study which demonstrated microUS is non-inferior to MRI for index lesion identification and potentially more sensitive for tumor extent. At smaller centers, microUS could be an inexpensive alternative to mpMRI for prostate cancer diagnosis. At larger centers, microUS could have a synergistic relationship with mpMRI to capitalize on the specificity of MRI for triaging patients for biopsy and the improved tumor margin identification of microUS during biopsy. However, to preserve diagnostic accuracy it is vital that microUS is used by well trained, expert reviewers. Due to the limited sample size of this study, a larger study is required to confirm these findings, but the conclusions presented here align closely with other microUS studies.

## Data Availability

The datasets generated during and analyzed during the current study are available from the corresponding author on reasonable request and approval of a data sharing agreement with UCLA.
